# Skin Lesions and Personal Protective Equipment in Health Care Workers From Lima, Peru, During the COVID-19 Pandemic: A Cross-sectional Study

**DOI:** 10.1093/ofid/ofaf509

**Published:** 2025-08-20

**Authors:** Carolina Coombes-Perez, Paula Arribas-Garcia, Amira Llerena-Delgado, Manuel Armando del Solar-Chacaltana, Rodrigo M Carrillo-Larco

**Affiliations:** Alberto Hurtado School of Medicine, Universidad Peruana Cayetano Heredia, Lima, Peru; Instituto de Medicina Tropical Alexander von Humboldt, Universidad Peruana Cayetano Heredia, Lima, Peru; Alberto Hurtado School of Medicine, Universidad Peruana Cayetano Heredia, Lima, Peru; School of Public Health and Administration, Universidad Peruana Cayetano Heredia, Lima, Peru; Alberto Hurtado School of Medicine, Universidad Peruana Cayetano Heredia, Lima, Peru; Infectious, Tropical and Dermatological Diseases Department, Hospital Cayetano Heredia, Lima, Peru; Hubert Department of Global Health, Rollins School of Public Health, Emory University, Atlanta, GA, USA

**Keywords:** COVID-19, occupation health, personal protective equipment, skin and soft tissue

## Abstract

**Background:**

During the COVID-19 pandemic, health care workers (HCWs) relied on personal protective equipment (PPE) to mitigate COVID-19 infections. However, prolonged use of PPE led to a risk of developing skin lesions (SLs).

**Methods:**

In this study, we evaluated the frequency, characteristics, and factors associated with SLs while using PPE. We surveyed 190 HCWs in 2 hospitals in Lima, Peru, between September 2020 and May 2021 using a convenience sampling method. Eligible participants were hospital personnel actively working on-site and using PPE. The questionnaire focused on reporting SLs related to PPE use.

**Results:**

The frequency of SLs among our sample of HCWs was 77%. The most frequent SLs were erythema (44%), comedones (18%), and erosions (15%), located most frequently on the nasal bridge (62%) and cheeks (28%). Of the surveyed HCWs, 39% reported using some form of prevention method to avoid SLs, with adhesive tape or bandages being the most common (58%). Working in the intensive care unit (adjusted prevalence ratio [aPR], 1.60; 95% CI, 1.11–2.29), working >12 hours per day (aPR, 1.64; 95% CI, 1.11–2.41), and experiencing burning sensations (aPR, 1.27; 95% CI, 1.05–1.55) or dryness (aPR, 1.39; 95% CI, 1.02–1.90) on the skin were associated with a higher likelihood of having SLs.

**Conclusions:**

By documenting the frequency of SL in our sample of HCWs while using PPE, we underscore the need for continuous biosafety monitoring to improve occupational well-being among HCWs.

During the COVID-19 pandemic, the Peruvian Ministry of Health established guidelines to prevent and control COVID-19 infections, including the mandatory use of personal protective equipment (PPE) for health care workers (HCWs) [1]. The recommended PPE included gowns or coveralls, surgical or N95 masks, safety glasses, face shields, head covers, and shoe covers.

The use of PPE during extended work hours and limited work breaks can lead to skin irritation, discomfort, and secondary skin infections [2, 3]. In fact, previous reports have shown that a prevalence of skin lesions related to PPE in HCWs ranges from 30% to 97% [2, 4–7]. The PPE used by HCWs can cause lesions through various mechanisms, such as the reduction of blood flow due to continuous pressure against the skin, weakening of the skin barrier by constant friction, and the disruption of the skin's normal microbiota from the use of alcohol-based disinfectants and the accumulation of water vapor from exhaled air and sweat [8]. The main PPE items associated with skin lesions include masks (surgical or N95), goggles, face shield, and gloves. Consequentially, the most anatomically damaged regions are the nasal bridge and cheeks [4, 6, 9, 10]. The skin lesions related to PPE use most described in the literature are pressure-related lesions that include erythema and erosion [3, 4]. Additionally, changes in skin pH, humidity, and temperature enabled by the use of PPE can condition inflammatory processes in patients with a history of skin conditions, such as contact dermatitis, eczema, acne, and rosacea [11–14]. Moreover, these potential dermatologic lesions might also compromise the HCWs’ ability to perform their duties properly due to discomfort and pain, further compromising their safety and that of the patients [15].

In resource-constrained countries such as Peru, which faced severe challenges during the COVID-19 pandemic, there is a notable lack of comprehensive data on PPE usage among HCWs, especially during the peak of the epidemic [16]. Peru experienced devastating consequences among HCWs, recording the third-highest mortality rate among doctors in Ibero-America during this period [17]. Studies have highlighted a high prevalence of skin lesions among HCWs [2, 4–6], which can lead to significant discomfort and potentially hinder proper PPE use—hence, increasing face touching, which can ultimately lead to higher risk of infectious disease transmission [18]. Understanding PPE usage during this time is critical to address gaps in occupational safety and preparedness. Thus, describing the profile of PPE usage, associated factors, and consequences is paramount to inform occupational safety guidelines and recommendations to improve safety and comfort and prevent skin lesions among HCWs using PPE for long hours. This is the case of HCWs in Peru and many other countries, where PPE continues to be a routine practice due to the high prevalence respiratory infections such as tuberculosis [19].

This study aims to identify the most common dermatologic lesions, their anatomic location, and associated skin symptoms experienced by HCWs working in a hospital setting using PPE during the COVID-19 pandemic. It also seeks to explore the management strategies employed to prevent and care for these lesions and examine preventive measures implemented under resource-constrained conditions. This study provides insights into improving PPE practices and safeguarding the skin health of HCWs in current and future public health emergencies.

## MATERIALS AND METHODS

### Study Design

This is an analytic cross-sectional study. We surveyed 190 HCWs from Arzobispo Loayza National Hospital (HNAL) and Cayetano Heredia Hospital (HCH) in Lima, Peru, between September 2020 and May 2021. This period largely represents the second wave of the COVID-19 pandemic in Peru.

### Settings

Lima has >10 million inhabitants, who represent 30% of the national population. The population density in the city amounts to 3879 persons/km^2^ [20]. HCH is a tertiary-level and national referral hospital situated at the north of the city in the second-most populated district [21]. During the pandemic, HCH was transformed into a COVID-19 hospital, increasing the number of beds from 362 to 464 [22]. HNAL is also a tertiary hospital and national referral hospital located in the city center. During the pandemic, HNAL increased intensive care unit (ICU) beds from 24 to 50 and allocated 279 hospitalization beds exclusively for patients with COVID-19 [23, 24]. Overall, HNC and HNAL provided comprehensive care for patients with COVID-19, and the HCWs in these institutions used PPE according to the national guidelines [1].

### Participants

Eligible participants were required to be hospital personnel, including attending physicians, resident physicians, nursing staff, technical staff, administrative staff, medical interns, physical therapy staff, and psychology staff from HNAL or HCH. Additionally, participants had to be actively working in their workplace using PPE throughout their working hours.

### Variables

Through self-reports via questionnaires, we collected data on skin lesions associated with PPE, which was our primary outcome of interest. Secondary outcomes included the anatomic site of the skin lesions associated with PPE, skin symptoms associated with PPE, treatment used for the skin lesions, and preventive practices to avoid skin lesions following the use of PPE. We also collected information on PPE use, sociodemographics, and history of skin conditions; these features were considered independent variables. The operational definition of all variables is described in Table 1.

**Table 1.** Operational Definition of Variables

Variable	Categorized
Outcome	
Skin lesions	*Primary lesions:* papule, plaque, macule, stain, ulcer, wheal, vesicle, blister*. Secondary lesions:* comedones, erosion, fissure, scales, maceration, erythema, excoriation. For definition of each lesion, see Supplementary material.
Site of skin lesion	Anatomic location of the skin lesion: nasal bridge, cheeks, forehead, limbs, trunk.
Skin symptoms	Subjective sensations in the skin, such as pruritus (itching), dryness, pain, burning.
Treatment for skin lesions	Treatments used to treat skin lesions: regenerative/hydrating cream, topical steroids, topical antibiotics, medicated cleanser, topical retinoids, systemic antibiotics, and other medication.
Preventive method	Device intended to prevent skin lesions caused by PPE: adhesive tape or bandage, silicone/hydrocolloid patch, and other devices.
Exposure	
Sex	Condition of the participant distinguished between male and female.
Age	Time in years from birth to the time of the survey: 20–29, 30–39, 40–49, 50–59, 60–69.
Hospital	The hospital where the participant worked: National Hospital Arzobispo Loayza or Hospital Cayetano Heredia.
Occupation	Occupation in the hospital: attending physician, resident physician, nursing staff, technical staff, administrative staff, medical intern, and others (physical therapy staff, psychology staff).
History of skin diseases	Chronic skin conditions previously diagnosed in the participant: acne, dermatitis, rosacea. Dermatitis included diagnoses such as allergic dermatitis, contact dermatitis, atopic dermatitis, and eczema.
Work area	Area of the hospital where the participant works: COVID-19 hospitalization, no COVID-19 hospitalization, emergency room, intensive care unit, outpatient clinic and other areas.
PPE	PPE is a set of biosecurity elements used to reduce the risk of virus transmission: surgical mask, N95 respirator, gown, goggles, shoe cover, apron, coveralls, head cover, face shield.
Hours using PPE	Number of hours per day that the respondent uses PPE in the hospital.
PPE usage days per week	Number of days per week that the respondent uses PPE in the hospital.
Personnel who indicated treatment for skin lesions	Person who indicated the medication to treat the reported skin lesions: self-medicated,^a^ pharmacy or drugstore, dermatologist, nondermatologist.

Abbreviation: PPE, personal protective equipment.

^a^Self-medication: the respondent, without being a dermatologist, indicated treatment for one’s own lesions.

We excluded hand skin lesions from our study, as glove use was not included in our PPE variable. We assumed that glove use followed standard practice in health care settings during the COVID-19 pandemic. Furthermore, we did not include handwashing habits in the analysis, as this was considered beyond the scope of our study.

### Data Collection

We developed the online questionnaire (Supplementary material), which focused on participants’ skin lesions related to PPE use during their time as HCWs amid the pandemic. The questionnaire was based on the studies by Lan et al and Jiang et al, where they studied skin lesions in HCWs during the early months of the COVID-19 pandemic in hospitals in China [3, 5]. Our questionnaire was developed in consultation with a dermatologist with >20 years of experience (M. A. S.-C.). To avoid potential misunderstandings and confusions, our questionnaire included figures of the skin lesions that were asked.

### Sample Size and Procedures

This study used a nonprobabilistic convenience sampling method. An estimated total of 6000 HCWs from both hospitals was obtained through the Transparency Portal of each hospital. Transparency Portal is a government website that releases public information from governmental institutions such as HCH and HNAL. A sample size was not calculated since the survey was designed to be applied in a census-like manner. This approach was chosen to maximize the number of responses and, consequently, the information collected. Additionally, the changing epidemiologic and operational situation (ie, staff experiencing work overload or leaving their positions) did not allow for the calculation of sample attrition adjustments. For this reason, we decided to invite all HCWs to participate to maximize sample size and diversity and to minimize losses due to death, resignation, or reassignment to home because of high-risk conditions for COVID-19.

Because the study was census based, we worked with the human resources office from each hospital, which had access to employees' email addresses and distributed virtual notices and invitations for this project. We did not have access to the email addresses. In addition, we used social media to reach HCWs, and for those who did not respond through these channels, we conducted in-person invitations for the online survey at the hospitals. Because we did not have access to the email addresses and did not collect any personal identifier, we did not keep track of how many participants were recruited from each channel.

### Statistical Analysis

Study data were entered via the Google Forms interface and analyzed in Stata version 18. Descriptive analysis was conducted presenting qualitative variables as frequencies and percentages. Bivariate analysis was performed with a χ^2^ test. For the crude model, we included the primary outcome and each covariate, using a generalized linear model Poisson family and log link with robust variance to calculate the prevalence ratio (PR) and corresponding 95% CIs. For the multivariate regression model, we performed a full model approach in the generalized linear model Poisson family and log link with robust variance. Variance inflation factor was used to detect multicollinearity in the multivariate regression model. *P* < .05 was considered statistically significant.

### Participant Consent Statement

Consent was obtained to participate in this study. The protocol and the informed consent were submitted to and approved by the Institutional Ethics Committee of Universidad Peruana Cayetano Heredia (SIDISI 202369) and the ethics committees of HNAL and HCH. The confidentiality of HCWs’ data was ensured by conducting anonymous surveys. This study posed no significant risk to participants.

## RESULTS

### Study Sample

The study population was 190 HCWs, who were mostly 30 to 39 years old (37.37%) and female (73.16%). Most of the survey respondents worked in the COVID-19 hospitalization area (31.58%) and worked 7 to 12 hours per day (78.42%), 1 to 3 days per week (41.05%).

Nearly half of the respondents reported having a previous skin condition (43.68%), the most common being dermatitis (66.67%), acne (31.33%), and rosacea (10.84%). Most HCWs used an N95 respirator (96.84%), disposable gown (78.95%), head cover (74.74%), eye gear (56.84%), and surgical masks (57.37%). Reported skin symptoms were skin dryness (62.11%), itchiness (54.21%), pain (33.16%), and burning sensation (13.68%; Table 2).

**Table 2.** Factors Associated With the Presence of Skin Lesions in Health Care Workers Using PPE (N = 190)

	Skin Lesions, No. (%)			
Variable	Total	Yes (n = 147)	No (n = 43)	*P* Value^a^
Female	139 (73.16)	111 (75.51)	28 (65.12)	.170
Hospital				.716
Hospital Cayetano Heredia	97 (51.05)	74 (50.34)	23 (53.49)	
Hospital Nacional Arzobispo Loayza	93 (48.95)	73 (49.66)	20 (46.51)	
Age, y				.084
20–29	47 (24.74)	40 (27.21)	7 (16.28)	
30–39	71 (37.37)	59 (40.14)	12 (27.91)	
40–49	27 (14.21)	18 (12.24)	9 (20.93)	
50–59	40 (21.05)	26 (17.69)	14 (32.56)	
60–69	5 (2.63)	4 (2.27)	1 (2.33)	
Occupation				**<.001**
Administration staff	12 (6.32)	3 (2.04)	9 (20.93)	
Medical intern	23 (12.11)	20 (13.61)	3 (6.98)	
Medical attending	68 (35.79)	50 (37.41)	13 (30.23)	
Medical resident	23 (12.11)	22 (14.97)	1 (2.33)	
Nurses	34 (17.89)	29 (19.73)	5 (11.63)	
Technicians	22 (11.58)	14 (9.52)	8 (18.60)	
Others	8 (4.21)	4 (2.27)	4 (9.30)	
Area in hospital				**.011**
COVID-19 hospitalization	60 (31.58)	49 (33.33)	11 (25.58)	
No COVID-19 hospitalization	51 (26.84)	44 (29.93)	7 (16.28)	
Emergency room	28 (18.60)	20 (13.61)	8 (18.60)	
Intensive care unit	18 (9.47)	16 (10.88)	2 (4.65)	
Outpatient clinic	12 (6.32)	7 (4.76)	5 (11.63)	
Other	21 (11.05)	11 (7.48)	10 (23.26)	
Average PPE use, h/d				.128
1–6	23 (12.11)	14 (9.52)	9 (20.93)	
7–12	149 (78.42)	119 (80.95)	30 (69.77)	
>12	18 (9.47)	14 (9.52)	4 (9.30)	
PPE use, d/wk				.479
1–3	78 (41.05)	57 (38.78)	21 (48.84)	
4–5	63 (33.16)	50 (34.01)	13 (30.23)	
6–7	49 (25.79)	40 (27.21)	9 (20.93)	
PPE used				
Eye gear/goggles	108 (56.84)	88 (59.86)	20 (46.51)	.120
N95 respirator	184 (96.84)	146 (99.32)	38 (88.37)	**<.001**
Protective apron	29 (15.26)	20 (13.61)	9 (20.93)	.240
Disposable gown	150 (78.95)	121 (82.31)	29 (67.44)	**.035**
Head cover	142 (74.74)	114 (77.55)	28 (65.12)	.099
Shoe cover	98 (51.58)	83 (56.46)	15 (34.88)	**.013**
Face shield	107 (56.32)	85 (57.82)	22 (51.16)	.439
Surgical masks	109 (57.37)	88 (59.86)	21 (48.84)	.198
Overalls	65 (34.21)	55 (37.41)	10 (23.26)	.085
History of skin conditions	83 (43.68)	72 (48.98)	11 (25.58)	**.007**
Type of previous skin conditions (n = 91)				
Acne	26 (31.33)	24 (33.33)	2 (18.18)	.313
Dermatitis	56 (66.67)	51 (69.86)	5 (45.45)	.109
Rosacea	9 (10.84)	7 (9.72)	2 (18.18)	.401
Skin symptoms				
Pain	63 (33.16)	56 (38.10)	7 (16.28)	**.008**
Burning sensation	26 (13.68)	24 (16.33)	2 (4.65)	.050
Itching	103 (54.21)	91 (61.90)	12 (27.91)	**<.001**
Dryness	118 (62.11)	107 (72.79)	11 (25.58)	**<.001**
Used a prevention method	71 (38.17)	65 (45.14)	6 (14.29)	**<.001**

Significant *P* values (<.05) are highlighted in bold.

Abbreviation: PPE, personal protective equipment.

^a^χ^2^ test.

### Prevalence of Skin Lesions

In our sample of HCWs using PPE, the prevalence of skin lesions was 77.37%. The most common lesions were erythema (43.75%), comedones (18.06%), erosions (14.58%), and maceration (8.33%), primarily affecting the nasal bridge (62.25%) and cheeks (28.48%). Hand skin lesions were not included as part of the analysis.

Of the respondents, 71 (48.30%) treated their skin lesions, with self-medication (46.48%) being the most common approach, followed by consultation with a dermatologist (42.25%). Regenerative or hydrating creams (43.02%) and topical steroids (26.74%) were the primary treatments. Additionally, 38.95% of participants reported using preventive measures against skin lesions, most frequently adhesive tape or bandages (58.11%; Table 3).

**Table 3.** Characteristics of Skin Lesions and Management (N = 190)

Variable	No. (%)
Presence of skin lesions (N = 190)	
Yes	147 (77.37)
No	43 (22.63)
Type of lesions (n = 144)	
Erythema	63 (43.75)
Comedones	26 (18.06)
Erosion	21 (14.58)
Maceration	12 (8.33)
Excoriation	8 (5.56)
Papules	4 (2.78)
Fissures	2 (1.39)
Blister	2 (1.39)
Macule	2 (1.39)
Scale	1 (0.69)
Skin spots	1 (0.69)
Plaque	1 (0.69)
Ulcer	1 (0.69)
Location of lesions (n = 151)	
Nasal bridge	94 (62.25)
Cheeks	43 (28.48)
Forehead	6 (3.97)
Chin	6 (3.97)
Received treatment (n = 147)	
Yes	71 (48.30)
No	76 (51.70)
Treatment prescription (n = 71)	
Self-medication	33 (46.48)
Dermatologist	30 (42.25)
Pharmacist	5 (7.04)
Doctor (not dermatologist)	3 (4.23)
Type of treatment (n = 86)	
Regenerative/hydrating cream	37 (43.02)
Topical steroids	23 (26.74)
Topical antibiotics	7 (8.14)
Medicated cleanser	7 (8.14)
Other	5 (5.81)
Topical retinoids	4 (4.65)
Systemic antibiotics	3 (3.49)
Prevention (N = 190)	
Yes	74 (38.95)
No	116 (61.05)
Prevention method (n = 74)	
Adhesive tape or bandage	43 (58.11)
Silicone/hydrocolloid patch	16 (21.62)
Others	15 (20.27)

### Associated Factors With Skin Lesions

In the full regression models, personnel working in the ICU (PR, 1.60; 95% CI, 1.11–2.29), working >12 hours per day (PR, 1.64; 95% CI, 1.11–2.41), reporting burning sensation in the skin (PR, 1.27; 95% CI, 1.05–1.55), and reporting skin dryness (PR, 1.39; 95% CI, 1.02–1.90) were more likely to experience skin lesions. Additionally, HCWs who worked 4 to 5 days per week (PR, 0.75; 95% CI, 0.61–0.92), worked 6 to 7 days per week (PR, 0.70; 95% CI, .51–.95), and used head covers (PR, 0.80; 95% CI, .66–.99) were less likely to experience skin lesions (Table 4).

**Table 4.** Crude and Multivariate Regression Analysis for the Presence of Skin Lesions (N = 190)

Characteristic	cPR	95% CI	*P* Value	aPR^a^	95% CI	*P* Value
Female	1.13	.93–1.38	.218	1.14	.90–1.45	.283
Age, y						
20–29	1 [Reference]			1 [Reference]		
30–39	0.98	.83–1.15	.769	0.88	.66–1.16	.362
40–49	0.78	.58–1.05	.102	0.78	.51–1.20	.260
50–59	0.76	.59–.99	**.040**	0.57	.31–1.03	.061
60–69	0.94	.60–1.48	.790	1.07	.51–2.26	.856
Occupation						
Administration staff	1 [Reference]			1 [Reference]		
Medical intern	3.48	1.29–9.41	**.014**	3.03	.64–14.32	.161
Medical attending	3.24	1.20–8.70	**.020**	2.79	.66–11.89	.165
Medical resident	3.83	1.43–1.26	**.008**	2.34	.53–1.35	.262
Nurses	3.41	1.26–9.20	**.015**	2.37	.53–1.55	.258
Technicians	2.55	.91–7.15	.076	2.45	.55–1.92	.241
Other	2.00	.09–.67	.259	1.07	.12–9.62	.953
Area in hospital						
COVID-19 hospitalization	1 [Reference]			1 [Reference]		
No COVID-19 hospitalization	1.06	.90–1.24	.509	0.92	.67–1.26	.605
Emergency room	0.87	.67–1.14	.320	1.15	.89–1.49	.285
Intensive care unit	1.09	.89–1.33	.414	1.60	1.11–2.29	**.011**
Outpatient clinic	0.71	.44–1.17	.182	1.33	.93–1.92	.122
Other	0.64	.42–.98	**.041**	1.11	.79–1.54	.551
Average PPE use, h/d						
1–6	1 [Reference]			1 [Reference]		
7–12	1.31	.94–1.84	.116	1.18	.83–1.70	.357
>12h	1.28	.85–1.93	.243	1.64	1.11–2.41	**.013**
PPE use, d/wk						
1–3	1 [Reference]			1 [Reference]		
4–5	1.09	.90–1.31	.382	0.75	.61–.92	**.006**
6–7	1.12	.92–1.35	.253	0.70	.51–.95	**.022**
PPE used						
Eye gear/goggles	1.13	.96–1.33	.134	0.92	.75–1.14	.459
N95 respirator	4.76	.79–28.67	.088	1.17	.62–2.22	.633
Protective apron	0.87	.68–1.13	.307	1.21	.94–1.57	.136
Disposable gown	1.24	.98–1.58	.079	1.08	.80–1.47	.612
Head cover	1.17	.95–1.44	.144	0.80	.66–.99	**.037**
Shoe cover	1.22	1.04–1.43	**.016**	1.20	.95–1.51	.128
Face shield	1.06	.91–1.25	.447	0.89	.65–1.22	.455
Surgical masks	1.11	.94–1.30	.213	1.19	.98–1.43	.073
Overalls	1.15	.99–1.33	.065	0.80	.63–1.03	.087
History of skin conditions	1.24	1.07–1.44	**.005**	…	…	…
Type of previous skin conditions						
Acne	1.10	.94–1.28	.257	1.20	.93–1.56	.168
Dermatitis	1.16	.94–1.43	.171	1.12	.91–1.37	.280
Rosacea	0.89	.62–1.27	.510	0.88	.62–1.25	.488
Skin symptoms						
Pain	1.24	1.08–1.43	**.003**	1.05	.88–1.27	.570
Burning sensation	1.23	1.07–1.42	**.004**	1.27	1.05–1.55	**.015**
Itching	1.37	1.16–1.63	**<.001**	0.95	.78–1.16	.596
Dryness	1.63	1.32–2.02	**<.001**	1.39	1.02–1.90	**.038**
Used a prevention method	1.31	1.14–1.51	**<.001**	0.98	.81–1.19	.873

Significant *P* values (<.05) are highlighted in bold.

Abbreviations: aPR, adjusted prevalence ratio; cPR, crude prevalence ratio; PPE, personal protective equipment.

^a^Adjusted by the following: age, sex, occupation, hospital area, average hours/day of PPE use, days per week using PPE, eye gear/goggles, N95 respirator, protective apron, disposable gown, head cover, shoe cover, face shield, surgical masks, overalls, medical history of dermatologic diseases, acne, dermatitis, rosacea, skin symptoms, prevention method.

## DISCUSSION

Our study demonstrates that among our sample of HCWs who use PPE in 2 hospitals in Lima, Peru, the frequency of skin lesions was 77.37%, which is on the higher end of the reported prevalences in literature [2, 4–7]. We found that working in the ICU, having work shifts >12 hours per day, and experiencing burning sensations or skin dryness were each associated with a higher likelihood of skin lesions. These findings demonstrate that skin lesions in our sample of HCWs are frequent and significant, reinforcing the need to integrate skin protection measures into occupational health policies to support the well-being of the HCWs.

Jiang et al reported 3 distinct types of frequent lesions: device-related pressure ulcers, moist-associated skin damage, and skin tears [5]. Our study showed similar findings with lesions, such as erythema, erosion, and maceration. However, we also found a high frequency of comedones, which may be attributed to the high proportion of participants who had a history of skin conditions. Furthermore, our study highlights that having a history of skin conditions is associated with the development of skin lesions, including comedones. Studies agree that using a mask and having a history of acne can aggravate or trigger flares of acne [25, 26]. As the increase of temperature and changes in the pH of the skin can favor changes in the microbiota, dermatologic conditions such as acne can result [26].

Our study shows that participants who worked shifts >12 hours per day and those who worked in the ICU were more likely to develop skin lesions. Results from other studies show that prolonged periods of PPE use have a detrimental effect on the skin due to constant friction and pressure of the PPE [27, 28]. For this reason, SECURE prevention guidelines and the NHS England establish that HCWs must relieve the mechanical pressure of the PPE at least every 2 hours, maintaining the skin clean and moisturized and using a skin protectant if the PPE is to be used for extended periods [29, 30]. Complying with these recommendations can be challenging in demanding and highly contagious hospital areas such as the ICU or during long-hour shifts, which could explain why in our study we found both factors associated with developing skin lesions. We also found that HCWs who worked 4 to 5 or 6 to 7 days per week were less likely to develop skin lesions. The reason may be that those working more days per week did so for fewer hours per day, thereby reducing the duration of PPE use each day.

Regarding management and prevention measures, in our study over half the HCWs received some kind of treatment, and of those who did receive treatment, half were self-medicated. Treatments ranged widely, from topical steroids to systemic antibiotics, underlining the severity of some lesions. Furthermore, the most reported preventive method of skin lesions was adhesive tape or bandages, which are free and easy to access in public hospitals but are not ideal as they can worsen the existing lesions or generate new ones. In contrast, various studies determine that hydrocolloids are the best way to prevent skin lesions [6, 31, 32], but these may not be well known or they may be expensive or hard to obtain in our study population, as only 21.62% (n = 16) used this preventive measure. In our sample, the results show that HCWs seem to manage skin lesions on their own and that there is an absence of an established pipeline for seeking treatment and accessing high-quality preventive care. This finding is supported by Hadjieconomou et al at the Princess of Wales Hospital, where the prevalence of pressure-related facial symptoms was low (4%). In this setting, staff were provided with hydrocolloid dressings in addition to instructions of daily use of an emollient as a skin barrier, highlighting the effectiveness of preventive interventions when properly implemented [33].

The strengths of our study include the involvement of a dermatologist in reviewing the questionnaire and the use of visual aids to facilitate accurate responses. Additionally, by including all HCWs in the hospital setting, rather than focusing solely on doctors and nurses, this study provides a more inclusive perspective on skin lesions among HCWs in general, which sets it apart from previous research [3, 5]. Moreover, we conducted the study in 2 major hospitals in Lima, thereby including a high-risk population.

Notwithstanding, this study has some limitations. First, despite the survey being designed with straightforward questions and visual aids, respondents may have made errors in identifying the type of lesions present. Second, since we used a nonprobabilistic convenience sampling method, a sampling bias may have occurred, as individuals with lesions and symptoms could have been more motivated to report their discomfort and therefore more inclined to complete the survey. This bias could have resulted in an overestimation of lesion frequency and may limit the generalizability of the results. Third, we did not include gloves as PPE in this study or describe handwashing habits, as we considered it outside the scope of our study; for this reason, we excluded results related to skin injuries located in the hands and instead focused on facial skin lesions. Fourth, our inclusion criteria, as stated in the Methods, were that participants had to be actively working in the workplace using PPE throughout their working hours. For this reason, we do not have an estimate of the frequency of skin lesions among HCWs not using PPE. Last, the cross-sectional design of the study precludes establishing causality between skin lesions and PPE usage.

## CONCLUSIONS

Our study highlights the high frequency of skin lesions among HCWs in Lima, Peru, during the COVID-19 pandemic, with 77.37% of participants reporting issues such as dryness, itchiness, erythema, comedones, erosion, and maceration. Factors such as extended shifts exceeding 12 hours, working in ICUs, and symptoms such as burning sensations or skin dryness were significantly associated with these lesions. These findings underscore the substantial dermatologic burden experienced by HCWs due to prolonged PPE use. Future longitudinal studies are warranted to explore causality, assess PPE design and materials, and develop preventive strategies to mitigate the impact of PPE-related skin conditions.

## Supplementary Material

ofaf509_Supplementary_Data

## Supplementary Data

Supplementary materials are available at *Open Forum Infectious Diseases* online. Consisting of data provided by the authors to benefit the reader, the posted materials are not copyedited and are the sole responsibility of the authors, so questions or comments should be addressed to the corresponding author.
