# Supplementary material for: Skin Lesions and Personal Protective Equipment in Health Care Workers From Lima, Peru, During the COVID-19 Pandemic: A Cross-sectional Study
Source: Open Forum Infect Dis. 2025 Aug 20;12(9):ofaf509. doi: 10.1093/ofid/ofaf509 (PMC12461874; doi:10.1093/ofid/ofaf509)
Supplement: ofaf509_Supplementary_Data [file ofaf509_supplementary_data.docx]

**Supplementary material**

**Annex 1: Survey**

| **GENERAL INFORMATION** |
| --- |
| Hospital   - Hospital Cayetano Heredia - Hospital Nacional Arzobispo Loayza |
| Sex   - Man - Woman |
| Age   - _________ |
| Occupation   - Medical attending - Medical resident - Nurses - Technicians - Administration staff - Medical intern - Other |
| Area in the hospital where you work   - Intensive Care Unit - COVID hospitalization - No COVID hospitalization - Outpatient clinic - Emergency room - Other |
| How many days a week do you work in an environment where personal protective equipment (PPE) is required?   - _________ |
| On average, how many hours per day do you use PPE (mask, goggles, gloves, apron)?   - _________ |
| During work hours, what type of protective equipment do you use? Please mark all that apply:   - Glasses/goggles - Face shield - N95 masks - Surgical masks - Apron - Gown - Head Cover - Show cover - Coverall |
| "What is the method you use most frequently for hand hygiene?"   - Hand sanitizer - Water and soap - Other:   Specify: ___________ |
| **MEDICAL HISTORY** |
| Do you have a history of previously diagnosed chronic dermatological conditions? Examples: hand eczema, atopic dermatitis, allergic dermatitis, acne, psoriasis, rosacea, etc.   - Yes - No |
| Please specify which dermatological condition you have: ___________ |
| **INJURIES CAUSED BY PERSONAL PROTECTIVE EQUIPMENT (PPE)** |
| Since you started caring for COVID-19 patients and using PPE, have you developed any skin injuries?   - Yes - No |
| Since you started caring for COVID-19 patients and using PPE, have you noticed any of the following characteristics on your skin?   - Itchiness - Dryness - Pain - Burning sensation - Redness or erythema |
| **LESIONS** |
| What type of lesion do you have? (Reference photos provided in Annex 2)   - Erythema: redness - Papule: small, solid lesion < 0.5 cm - Plaque: large, elevated lesion with a flat surface - Macule: non-palpable lesion <1 cm that varies in pigmentation - Spot: non-palpable lesion >1 cm - Vesicle: elevated lesion with liquid content <0.5 cm - Blister: elevated lesion with liquid content >0.5 cm - Wheal (hive): a raised, erythematous, edematous papule or plaque. Disappears within 24-48 hours. - Ulcer: loss of epidermis with dermis loss, forms a scar - Scales: excessive accumulation of the stratum corneum that flakes off - Maceration: weakening and decomposition of the skin due to prolonged exposure to moisture - Fissure: a lesion caused by deep splitting of the skin extending into the dermis - Erosion: loss of the epidermis without related dermal loss - Excoriation: erosion caused by scratching - Comedones: the main sign of acne |
| Where did the lesion occur?   - Nasal bridge - Hands - Cheeks - Forehead - Extremities - Trunk - Other: |
| Have you had any other lesions?   - Yes - No |
| Please describe the type of lesion and its location _________________ |
| **TREATMENT** |
| Have you used any treatment for the lesions?   - Yes - No |
| Who recommended the treatment?   - Self-medicated - Pharmacy or drugstore - Dermatologist - Other: |
| If the answer was Yes, what treatment have you used? Specify: _________________ |
| **PREVENTION** |
| Have you used any product to prevent skin damage? For example: creams, patches, band-aids, cotton, others   - Yes - No - Other: |
| What product have you used? Specify: _________________ |

**Annex 2**

| Erythema: (1)  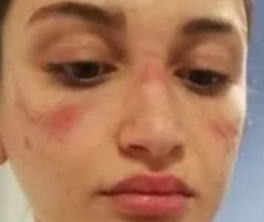 | Vesicule: (2)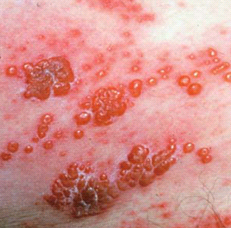 | Maceration: (3)  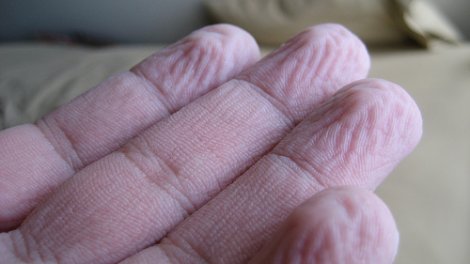 |
| --- | --- | --- |
| Papule: (2)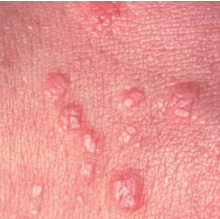 | Blister: (2)  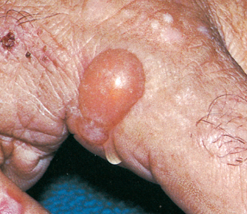 | Fissure: (2)  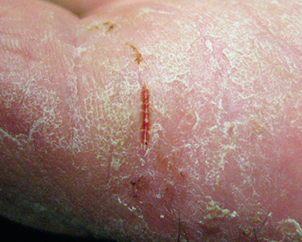 |
| Plaque: (2)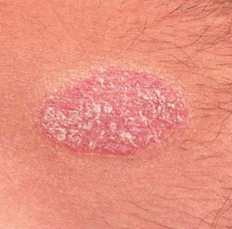 | Wheal (hive): (2)  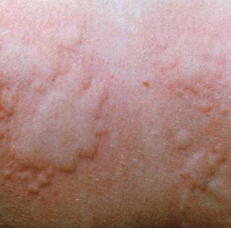 | Erosion: (1)  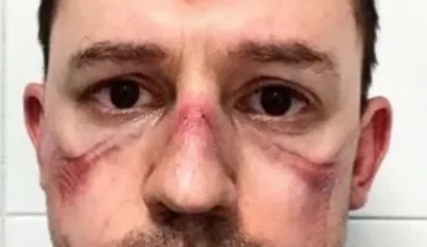 |
| Macule: (2)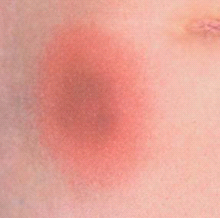 | 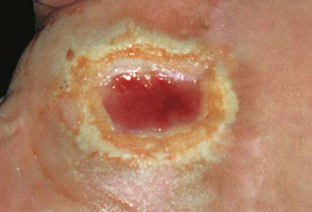Ulcer: (2) | Comedones: (2)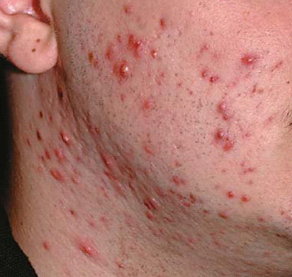 |
| Spot: (2)  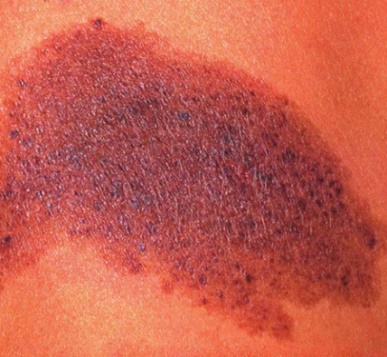 | Scales: (2)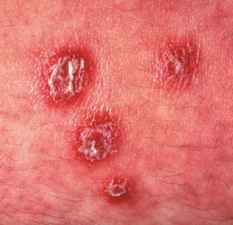 | Excoriation: (2)  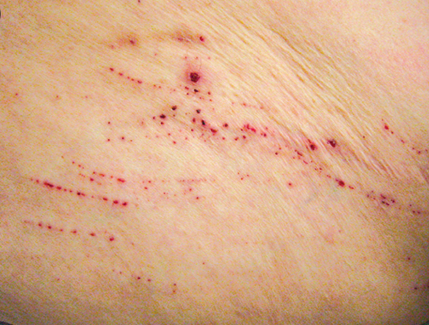 |

**Pictures:**

1. Mag R. Coronavirus: This is what the faces of healthcare professionals look like in their tireless fight against COVID-19 [Internet]. Mag. 2020. Available at: <https://mag.elcomercio.pe/historias/coronavirus-asi-se-ve-el-rostro-de-los-medicos-que-luchan-contra-el-covid-19-sin-descanso-orthocoronavirinae-nnda-nnrt-noticia/>
2. Wolff K, Johnson RA, Saavedra AP, Roh EK. *Fitzpatrick’s Color Atlas and Synopsis of Clinical Dermatology* - 6th ed. [Internet]. McGraw-Hill; 2017. Available at: <http://thuvienso.vanlanguni.edu.vn/handle/Vanlang_TV/14200>
3. Why (and How) Your Skin Wrinkles Underwater – Proslogion [Internet]. Available at: <https://blog.drwile.com/why-and-how-your-skin-wrinkles-underwater/>
